# Titanium salts tested in reconstructed human skin with integrated MUTZ‐3‐derived Langerhans cells show an irritant rather than a sensitizing potential

**DOI:** 10.1111/cod.13666

**Published:** 2020-08-06

**Authors:** Charlotte T. Rodrigues Neves, Sander W. Spiekstra, Niels P. J. de Graaf, Thomas Rustemeyer, Albert J. Feilzer, Cees J. Kleverlaan, Susan Gibbs

**Affiliations:** ^1^ Department of Molecular Cell Biology and Immunology, Amsterdam University Medical Centre, Amsterdam Infection and Immunity Institute Vrije Universiteit Amsterdam Amsterdam The Netherlands; ^2^ Department of Oral Cell Biology, Academic Centre for Dentistry (ACTA) University of Amsterdam and Vrije Universiteit Amsterdam Amsterdam The Netherlands; ^3^ Department of Dermatology Amsterdam University Medical Centre (location AMC) Amsterdam The Netherlands; ^4^ Department of Dental Materials Science, Academic Centre for Dentistry (ACTA) University of Amsterdam and Vrije Universiteit Amsterdam Amsterdam The Netherlands

**Keywords:** allergy, in vitro, irritant, Langerhans cell, organotypic, reconstructed human skin, sensitizer, titanium

## Abstract

**Background:**

The nature of clinically related adverse reactions to titanium is still unknown.

**Objective:**

To determine whether titanium salts have irritant or sensitizing potential in a reconstructed human skin (RHS) model with integrated Langerhans cells (LCs).

**Methods:**

RHS‐LCs (ie, reconstructed epidermis) containing primary differentiated keratinocytes and CFSE^+^CD1a^+^‐LCs generated from the MUTZ‐3 cell line on a primary fibroblast‐populated collagen hydrogel (dermis) were topically exposed to titanium(IV) bis(ammonium lactato)dihydroxide (TiALH). LC migration and plasticity were determined.

**Results:**

TiALH resulted in CFSE^+^CD1a^+^‐LC migration out of the epidermis. Neutralizing antibodies to CCL5 and CXCL12 showed that LC migration was CCL5 and not CXCL12 mediated. LCs accumulating within the dermis after TiALH exposure were CFSE^+^Lang^+^CD68^+^ which is characteristic of a phenotypic switch of MUTZ‐LC to a macrophage‐like cell. Furthermore, TiALH did not result in increased interleukin (IL)‐1β or CCR7 messenger RNA (mRNA) in the dermis, but did result in increased IL‐10 mRNA. In addition, monocultures of MUTZ‐LCs failed to increase LC maturation biomarkers CD83, CD86, and CXCL‐8 when exposed to noncytotoxic concentrations of four different titanium salts.

**Conclusion:**

These results classify titanium salts as irritants rather than sensitizers and indicate that titanium implant‐related complaints could be due to localized irritant‐mediated inflammation arising from leachable agents rather than a titanium metal allergy.

## Graphical abstract

Titanium scores as irritant not sensitizer.

- In vitro reconstructed human skin model with integrated Langerhans cells (RHS‐LCs) can be used for determining sensitizer or irritant potential of metals.
- Titanium(IV) bis(ammonium lactato)dihydroxide (TiALH) resulted in CFSE^+^CD1a^+^Lang^+^CD68^+^LC migration out of the epidermis, indicating a phenotypic switch of a macrophage‐like cell. LC migration was CCL5 (irritant) and not CXCL12 (sensitizer) mediated.
- Results classify titanium salts as irritants rather than sensitizers and indicate that titanium implant‐related complaints could be due to localized irritant‐mediated inflammation rather than titanium metal allergy.

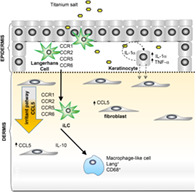

**Abbreviations**
C_12_H_28_O_4_Ti: titanium tetraisopropanolate
CaO_3_Ti: calcium titanium oxide
CFSE: carboxyfluorescein succinimidyl ester
DSA_05_: chemical dose per skin area leading to a sensitization incidence of 5%
MUTZ‐LCs: MUTZ‐3‐derived Langerhans cells
RHE: reconstructed human epidermis
RHS‐LCs: reconstructed human skin with integrated Langerhans cells
TiO_2_: titanium dioxide

## 1 INTRODUCTION

Titanium or titanium alloys are extensively used in medical devices (eg, orthopaedic surgery and dentistry) as an alternative for nickel, chrome, and cobalt to which many patients are allergic.^1^ Furthermore, titanium is being generously incorporated into jewellery and as a white pigment (TiO_2_) in personal health care products such as toothpaste, cosmetics, and sunscreen,^2^, ^3^ and therefore daily human exposure to titanium is increasing.^4^, ^5^ Titanium is thought to be an inert, hypo‐allergenic material and is often chosen as an implant material due to its high biocompatibility, corrosion resistance, and strength.^6^, ^7^ However, titanium‐based implants can release particles and ions into surrounding tissues and bodily fluids.^8^, ^9^, ^10^ Furthermore, clinical experience indicates a relevant number of adverse reactions related to titanium implants. Some of these adverse reactions suggest that titanium may be a contact sensitizer which can elicit a type IV delayed hypersensitivity reaction.^11^, ^12^, ^13^ Reported symptoms of a suspected titanium allergy in addition to aseptic loosening of implants are dermatitis, stomatitis, chronic inflammation in adjacent tissue, impaired wound healing, and acne‐like facial inflammation.^14^, ^15^ Yet, the existence of a true titanium allergy has been much debated.^15^

According to the EU Medical Devices legislation that came into effect in 2017, manufactures have to comply with higher standards of quality and safety for medical devices to meet common safety concerns. With regard to testing sensitization potential of metal ions derived from medical devices, data from animal or human studies performed under standardized conditions are extremely limited. Metals are difficult chemicals to test in the mouse local lymph node assay as well as in human assays assessing chemical dose per skin area leading to a sensitization incidence of 5% (DSA_05_); no observed effect level; and lowest observed effect level, and are generally tested as metal salts.^16^, ^17^ Therefore, currently titanium remains as an unclassified chemical with regard to sensitization and irritation potential.^18^

In the past, human in vitro methods have been developed to identify contact sensitizers, such as Direct Peptide Reactivity Assay (OECD‐TG 442C), KeratinoSens (OECD‐TG 442D), and human cell line activation test, to replace animal models, such as the local lymph node assay.^19^, ^20^ Many new in vitro methods varying in complexity to identify sensitizers from nonsensitizers and irritants are in development.^21^ As described in the adverse outcome pathway (AOP) for skin sensitization,^19^ keratinocytes (KCs) play a key role in sensitization and activation of the immune response. They are able to trigger an inflammatory response through the release of inflammatory cytokines (key events 1 and 2 of the AOP described in the OECD test number 168). Among these secreted cytokines, interleukin (IL)‐18 has been shown to play a pivotal role in allergic contact dermatitis (sensitization) but not in irritant contact dermatitis by inducing the migration of dendritic cells (DCs) to the lymph nodes (key event 3), where they present the haptenized proteins to naïve T cells, resulting in T‐cell priming and memory^22^, ^23^ and also promote a helper type 1‐type immune response (key event 4) by stimulating the secretion of proinflammatory mediators such as tumour necrosis factor (TNF)‐α, CXCL8, and interferon‐γ.^23^, ^24^ In the past, we have developed a reconstructed human epidermis (RHE) model to identify contact sensitizers from nonsensitizers and assess sensitizer potency^25^ by measuring IL‐18 release into the culture medium after topical exposure of chemicals to the stratum corneum. IL‐18 has proven to be a relevant biomarker for assessing sensitizing potential of chemicals in in vitro assays.^25^, ^26^, ^27^ An expanded study with the RHE model showed that exposure to known sensitizing metal salts, such as potassium dichromate, chromium(III) chloride, nickel(II) chloride hexahydrate, nickel(II) sulfate hexahydrate, gold(I) chloride, sodium aurothiosulfate(I), cobalt(II) chloride, mercuric(II) chloride, and copper(II) sulfate, in contrast to chemical sensitizers, resulted in no increase or only a small increase in IL‐18 production in RHE.^18^ Specifically, the four titanium salts, namely, titanium(IV) isopropoxide, titanium(IV) bis(ammonium lactate) dihydroxide (TiALH), titanium(IV) oxide, and calcium titanate that were tested failed to result in an increase in IL‐18 release into the culture medium.^18^ The reasons for this low IL‐18 release after metal exposure are currently unknown; however, it is important to remember that the RHE IL‐18 assay only identifies a type IV hypersensitivity reaction and chemicals triggering humoral hypersensitivity responses (types II and III) are likely to be negative.^17^, ^18^

Because the RHE model lacks Langerhans cells (LCs), which are key players in the AOP for sensitization, in this study we used a more advanced model that integrates LCs into reconstructed human skin (RHS) to investigate further the sensitization or irritant potential of titanium. The human acute myeloid leukaemia cell line MUTZ‐3 can be differentiated in a transforming growth factor (TGF)‐β‐dependent fashion into LCs (MUTZ‐LCs), expressing langerin and bearing LC‐associated Birbeck granules.^28^, ^29^ In monoculture, upon stimulation with contact sensitizers, MUTZ‐LCs upregulate characteristic maturation markers, such as CD83, CD86, and CXCL8, and acquire the ability to migrate towards CXCL12, thus closely resembling their in vivo counterparts.^30^, ^31^, ^32^ When MUTZ‐LCs are incorporated into RHS, they show the same differential migration and phenotypic plasticity after allergen or irritant exposure as is seen in native skin.^33^, ^34^ Upon allergen exposure MUTZ‐LCs take on a mature phenotype, as observed by an increase in IL‐1β messenger RNA (mRNA)^35^ and increase of expression of surface maturation markers CD83 and CD86.^32^ At the same time, the expression of receptors involved in homing to the local lymph nodes, CXCR4 and CCR7, becomes upregulated and MUTZ‐LCs migrate in a CXCL12‐dependent manner into the dermis.^31^, ^34^ Upon irritant exposure MUTZ‐LCs do not mature but migrate in a CCL5‐dependent manner into the dermis, where they undergo a phenotypic change into a macrophage‐like cell (CD1a^−^/CD68^+^) under the influence of IL‐10.^36^ The advantages of using the MUTZ‐3 cell line above primary DCs derived from monocytes is realized when appreciating the logistics, complexity, and reproducibility of the model as well as the scalability. Therefore, our immune‐competent RHS‐LC is a physiologically relevant human model to distinguish chemical sensitizers from irritants in vitro, and to investigate titanium salts further.

In this study we used the RHS‐LC model and MUTZ‐LCs to determine whether titanium salts have sensitizer or irritant potential and in doing so addressed key events 1, 2, and 3 of the AOP (OECD test number 168, 2012) for skin sensitization, namely, epidermal penetration, cytokine secretion, and DC activation.

## 2 MATERIALS AND METHODS

### 2.1 Cell culture

Human neonatal foreskin was obtained after informed consent from healthy donors undergoing routine surgical procedures and was used anonymously, in compliance with the VU University Medical Centre, Amsterdam UMC ethical guidelines and the “Code for Proper Use of Human Tissues” as formulated by the Dutch Federation of Medical Scientific Organizations (www.fedora.org). Epidermal KCs and dermal fibroblasts were isolated and cultured as previously described^37^, ^38^ and grown until 80% to 90% confluency.

MUTZ‐3 progenitor cells (Deutsche Sammlung von Mikroorganismen und Zellkulturen, Braunschweig, Germany) were maintained as previously described^28^ until maximally passage number 35. MUTZ‐3 progenitor cells were differentiated into LCs (MUTZ‐LCs) in minimal essential medium‐alpha (Gibco, Grand Island, New York) supplemented with 20% vol/vol heat‐inactivated calf serum (HyClone laboratories, Logan, Utah), 1% penicillin‐streptomycin, 2 mM l‐glutamine (Gibco), 50 μM 2‐mercaptoethanol (Merck, Whitehouse Station, New York), 100 ng/mL recombinant human granulocyte macrophage‐colony stimulating factor (BioSource International, Camarillo, California), 10 ng/mL TGF‐β (BioVision, Mountain View, California), and 2.5 ng/mL TNF‐α (Strathmann Biotec, Hamburg, Germany) for 7 days at 37°C, 5% CO_2_, and 95% humidity.

*Construction of RHS‐LC*: MUTZ‐LCs were labelled with carboxyfluorescein succinimidyl ester (CFSE; Thermo Fisher, Waltham, Massachusetts) before incorporation into the RHS as previously described.^32^ RHS‐LCs were constructed by preparing a fibroblast (passage number maximally 2) populated collagen hydrogel (collagen isolated from rat tails) and coseeding KCs passage number 0 or passage number 1 (0.5 × 10^6^ cells) and CFSE‐labelled MUTZ‐LCs (1 × 10^6^ cells) on top of the hydrogel as previously described.^33^ The RHS‐LCs were cultured submerged in KC I medium (Dulbecco's modified Eagle medium; Lonza, Basel, Switzerland)/Ham's F‐12 (Gibco) (3:1) containing 1% UltroserG (BioSepra, Cergy‐Saint‐Christophe, France), 1% penicillin‐streptomycin (Gibco), 1 μmol/L hydrocortisone, 1 μmol/L isoproterenol, 0.1 μmol/L insulin and supplemented with 1 ng/mL KGF for 4 days at 37°C at 7.5% CO_2_. After 4 days the RHS‐LCs were lifted to the air‐liquid interface and cultured for 10 days in KC II medium containing 1 × 10^−5^ M l‐carnitine, 1 × 10^−2^ M l‐serine, and 50 μg/mL ascorbic acid as well as 2 ng/mL KGF before chemical exposure. All additives were purchased from Sigma‐Aldrich (St. Louis, Missouri) unless stated otherwise. Each RHS‐LC batch was constructed from two or three pooled foreskin donors using MUTZ‐3 (maximum passage 35) and donor‐matched KCs (maximum passage P1) and fibroblasts (maximum P2). The number of independent experiments, each representing a different RHS‐LC batch, is indicated in the figure legends.

### 2.2 Chemical exposure

MUTZ‐LCs were cultured in a 24‐well plate (3.0 × 10^5^ cells/mL/well) and exposed to four different titanium salts, namely, TiO_2_ (CAS no. 13463‐67‐7), CaO_3_Ti (CAS no. 12049‐50‐2), C_12_H_28_O_4_Ti (CAS no. 546‐68‐9), and TiALH (CAS no. 65104‐01‐5); nickel sulfate (NiSO_4_; CAS no. 10101‐97‐0); H_2_O or a cytokine maturation cocktail (CMC) containing 100 ng/mL IL‐6, 25 ng/mL IL‐1β, 50 ng/mL TNF‐α, and 1 μg/mL PGE_2_ for 16 hours at 37°C at 5% CO_2_. H_2_O was used as vehicle. All chemicals were obtained from Sigma‐Aldrich. Cytokines were purchased from Miltenyi Biotec (Bergisch Gladbach, Germany).

RHS‐LCs were placed in 1.5 mL of hydrocortisone‐free KC II medium. Finn Chamber filter paper disks of 11‐mm diameter (Epitest, Tuusula, Finland) were impregnated with vehicle (H_2_O), TiALH or NiSO_4_ dissolved in H_2_O as described previously,^25^ or CMC. Chemical‐ or vehicle‐impregnated disks were applied topically to the stratum corneum of RHS‐LCs for 24 hours at 37°C, 7.5% CO_2._ For the blocking experiments, previously established optimal blocking concentrations of 7 μg/mL goat antihuman CCL5 (AF‐278‐NA; R&D systems, Minneapolis, Minnesota), 7 μg/mL goat antihuman CXCL12 (AF‐310‐NA, R&D systems), or 7 μg/mL polyclonal goat immunoglobulin G (IgG)G isotype antibody (6‐001‐F, R&D systems) were added to the culture medium 2 hours prior to chemical exposure.^31^, ^36^

### 2.3 Determination of RHS‐LCs viability

The MMT assay measuring mitochondrial activity, which is representative of cell viability, was performed as previously described.^39^ Punch biopsies of 3 mm^2^ were placed on top of 200 μL MTT (Sigma) dissolved in phosphate‐buffered saline (PBS; 5 μM) in a 96‐well plate for 2 hours. RHS‐LC biopsies were then transferred to a new 96‐well plate and incubated overnight in the dark, at room temperature with 0.5 mL isopropanol (Merck). The next day, absorbance was measured at 570 nm with a Mithras LB940 spectrophotometer. Results are expressed relative to unexposed controls.

### 2.4 Histology

RHS‐LCs were fixed in 4% formaldehyde and conventionally embedded in paraffin. Paraffin sections of 5‐μm thickness were cut, deparaffinized, and rehydrated in preparation for morphological analysis (haematoxylin and eosin staining).

### 2.5 Quantitation of MUTZ‐LCs in epidermal sheet

After chemical exposure, the epidermis of the RHS‐LCs was separated from the hydrogel using forceps. Subsequently, the epidermal sheet was submerged in fluorescence‐activated cell sorting (FACS) buffer (PBS supplemented with 0.1% bovine serum albumin and 0.1% sodium azide) for 1 hour at 4°C (maximum overnight) with 100 μL/mL phycoerythrin (PE)‐labelled antigen CD1a (BD Pharmingen, San Diego, California) in FACS buffer and examined with a fluorescence microscope (Nikon Eclipse 80i, G‐2a Ex510‐560, DM575, BA590). The density of MUTZ‐LCs was determined by calculating CFSE^+^/CD1a^+^ cells based on their fluorescence intensity with NIS‐Elements software (Nikon Instruments Europe, Amsterdam, The Netherlands).

### 2.6 Antibodies and flow cytometry

Migration of MUTZ‐LCs from the epidermis in RHS and maturation of MUTZ‐LCs were assessed by flow cytometry. Cell staining was performed using mouse antihuman CD1a‐PE (IgG1; BD Pharmingen), intracellular CD68‐PE (kit 556 079, IgG2b, κ; BD Pharmingen), langerin APC (IgG1; Miltenyi Biotec), CD86‐FITC, and CD83‐PE (IgG1; BD Biosciences). Isotype controls to assess nonspecific binding were mouse IgG1‐PE, IgG2b, κ (BD Pharmingen), and mouse IgG2a‐FITC (Miltenyi Biotec). Cells were washed and resuspended in FACS buffer (PBS supplemented with 0.1% bovine serum albumin and 0.1% sodium azide) and incubated for 30 minutes at 4°C in the presence of the antibodies. Subsequently the cells were resuspended in the same FACS buffer with an abundance of 123count eBeads (Thermo Fisher) before analysis using a FACSCalibur flow cytometer (Beckton Dickinson, San Jose, California). In addition, a propidium iodide (GIBCO) staining was performed to confirm the viability of MUTZ‐LC monocultures. All data were analyzed using CellQuest Pro FACS analysis software (BD Pharmingen).

### 2.7 Quantification of MUTZ‐LC in collagen hydrogel

MUTZ‐LCs (and fibroblasts) were isolated from the collagen hydrogel using the gentleMACS Dissociator (Miltenyi Biotec). FACS buffer (2 mL) was added to the hydrogel and after dissociation the suspension was filtered using a 100‐μm cell strainer (Corning, Sigma‐Aldrich). An excess of 123count eBeads (Thermo Fisher) was added to the harvested cells that were subsequently stained with CD1a‐PE (IgG1; BD Pharmingen) and langerin APC (IgG1; Miltenyi Biotec) before analysis with an FACSCalibur flow cytometer using CellQuest Pro software (BD Biosciences).

### 2.8 Real‐time PCR

After removal of the epidermis with fine forceps, total RNA was isolated from the hydrogel and real‐time polymerase chain reaction (RT‐PCR) analysis was performed as previously described.^33^ cDNA was amplified by PCR using the following primer kits: RT^2^ qPCR Primer Assay for human IL‐1β, CCR7, IL‐10, and housekeeping genes HPRT and GAPDH (OriGene Technologies, Rockville, Maryland).

### 2.9 Statistical analysis

Statistical analysis in the different experimental conditions was performed using one‐way analysis of variance followed by Friedman's multiple comparisons test or Mann‐Whitney *U* test, as indicated in the figure legends, by GraphPad Prism version 7.00 for Microsoft Windows (GraphPad Software, La Jolla, California); *P* < .05 was considered significant. The number of independent experiments, each representing a different MUTZ‐LC or RHS‐LC batch, is indicated in the figure legends.

## 3 RESULTS

### 3.1 Expression of sensitization biomarkers CD83, CD86, and CXCL8 after MUTZ‐LC exposure to titanium salts

**FIGURE 1:**
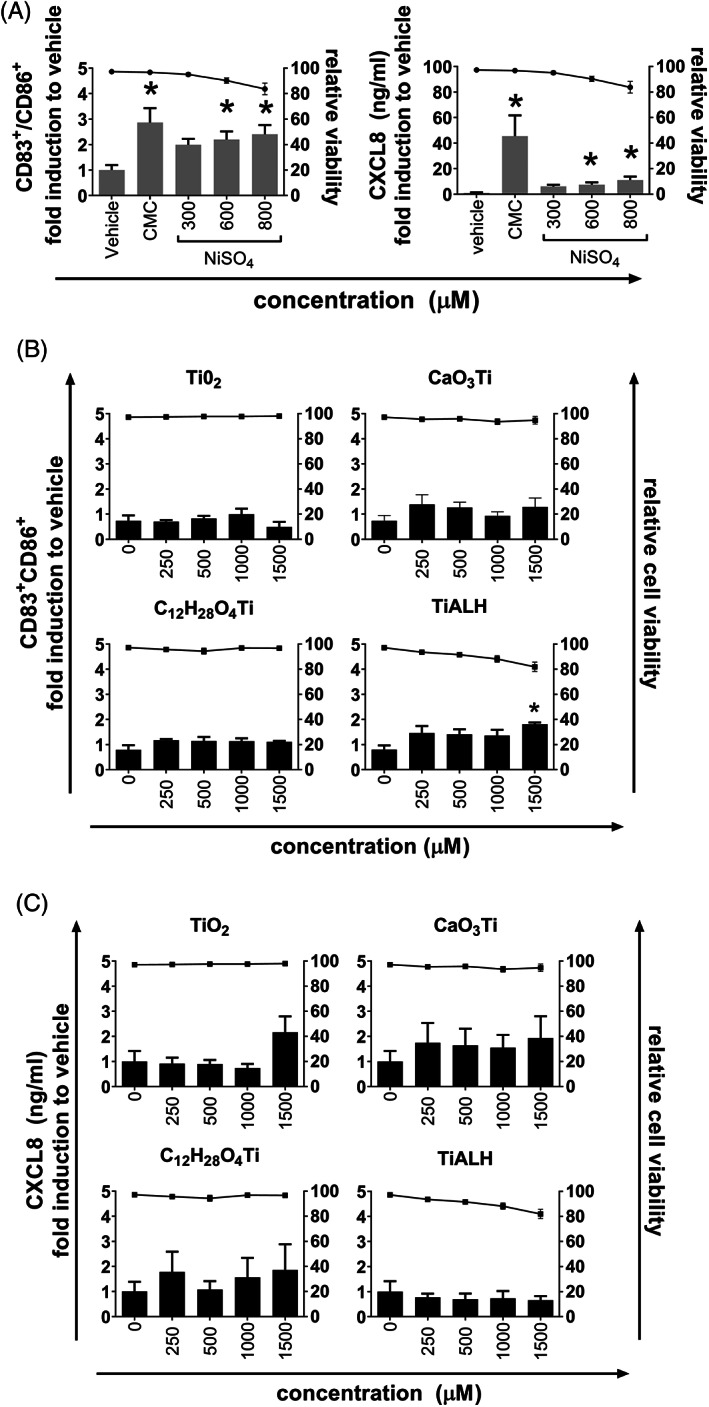
Chemical exposure of MUTZ‐LCs. MUTZ‐LCs were exposed to chemicals or vehicle (water) for 16 hours as described in the “Materials and Methods” section. CD83/CD86 double‐positive cells were quantified by flow cytometry using Flow‐Count fluorospheres and CD83‐PE and CD86‐FITC antibodies; CXCL8 secretion was determined by ELISA (bars). Relative viability compared with vehicle‐exposed MUTZ‐LCs was determined by propidium iodide uptake (flow cytometry; dots). (**A**) MUTZ‐LC maturation by NiSO_4_ and cytokine maturation cocktail (CMC). (**B**) The percentage of CD83/CD86 double‐positive cells after titanium salt exposure. (**C**) CXCL8 secretion after titanium salt exposure. The figures depict the average of four individual experiments performed in duplicate ± SEM. *P < .05 calculated using the Friedman multiple comparisons test. FITC, fluorescein isothiocyanate; LC, Langerhans cell; PE, phycoerythrin

Previously we have shown that MUTZ‐LCs increased surface marker expression of CD83 and CD86 and increased secretion of CXCL8 when exposed to sensitizers, but not when exposed to nonsensitizers.^32^ Therefore, to determine the in vitro sensitization potential of titanium in this assay, MUTZ‐LCs were exposed to four different titanium salts and compared with MUTZ‐LCs exposed to the positive controls, NiSO_4_ and a cytokine maturation cocktail (CMC). First, cytotoxicity of the salts was determined by the extent of propidium iodide uptake. TiALH was the only titanium derivative which resulted in mild cytotoxicity at the highest concentration tested (18% ± 7.4% propidium iodide‐labelled cells at 1500 μM; Figure 1). A similar degree of cytotoxicity was observed with NiSO_4_ at the highest tested concentration of 800 μM; while CMC was not cytotoxic (Figure 1A). Exposure to NiSO_4_ and CMC both significantly increased the expression of surface markers CD83 and CD86 and secretion of CXCL8 in MUTZ‐LCs, confirming the ability of MUTZ‐LCs to mature when exposed to relevant stimuli (Figure 1A). By contrast, only the highest concentration of TiALH (1500 μM) was able to increase expression of CD83 and CD86 (Figure 1B). None of the titanium salts were able to increase CXCL8 secretion (Figure 1C).

### 3.2 Epidermal to dermal migration of MUTZ‐LCs in RHS after titanium(IV) bis(ammonium lactato)dihydroxide (TiALH) exposure is CCL5 dependent

**FIGURE 2:**
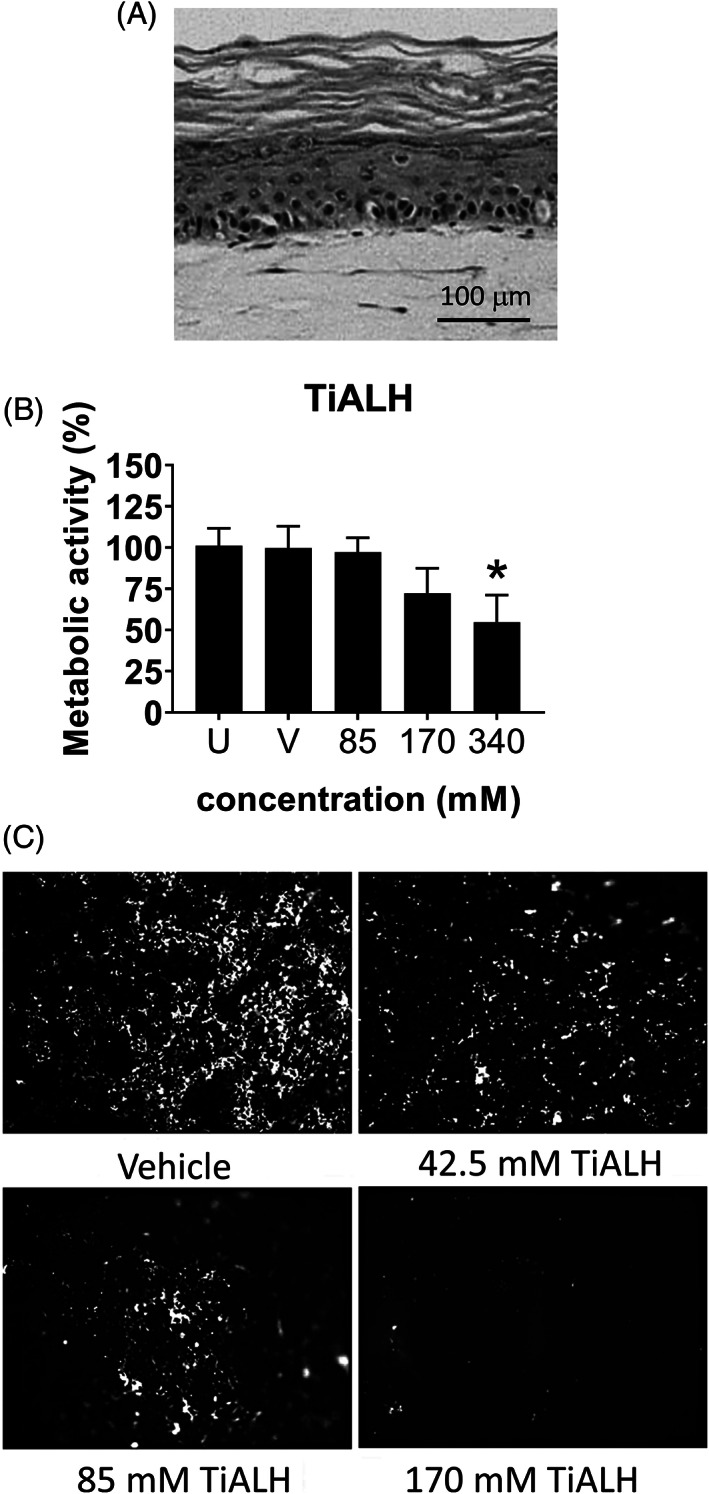
MUTZ‐LCs migration from RHS epidermis after exposure to titanium(IV) bis(ammonium lactato)dihydroxide. RHS‐LCs were unexposed (U), exposed to H_2_O vehicle (V), or TiALH for 24 hours. (**A**) Histology of RHS‐LCs (haematoxylin and eosin staining of 5‐μm paraffin‐embedded tissue section). (**B**) Metabolic activity, corresponding to viability, was determined by MTT assay. The data depict the average of four individual experiments performed in duplicate (RHS‐LC ± SEM). *P < .05 calculated using the Friedman multiple comparisons test. (**C**) Epidermal sheets isolated from RHS‐LCs stained with anti‐CD1a‐PE are shown. Florescence intensity (light) shows the presence of MUTZ‐LCs in the epidermal sheets. LC, Langerhans cell; PE, phycoerythrin; RHS, reconstructed human skin; SEM, standard error of the mean; TiALH, titanium(IV) bis(ammonium lactato)dihydroxide

Because TiALH was the only titanium salt that resulted in a slight decrease in cell viability which may correlate with an irritant potential, and a slight increase in MUTZ‐LC maturation markers which may correlate with a sensitization potential, this salt was used for further experiments to investigate irritant and sensitization properties in RHS‐LCs (Figure 2). First, RHS‐LCs were topically exposed to a concentration range of TiALH to identify the lowest noncytotoxic concentration of the salt, and to determine whether titanium could initiate MUTZ‐LCs migration out of the epidermis at relatively noncytotoxic concentrations (≤25%; Figure 2). MUTZ‐LCs were detected as CD1a^+^ immune‐stained cells with dendrites distributed throughout the multilayered (three dimensional) epidermal sheets—observed by brightest cells being at the surface and less bright cells being at increasing depths within the sheet (due to technical issues it was not possible to immune stain epidermal sheets with langerin). The highest concentration of titanium tested (340 mM) resulted in 54.7% ± 16.5% reduction in metabolic activity, and therefore this concentration was no longer included in further experiments (Figure 2B). Compared with vehicle and unexposed RHS‐LCs, 85 and 170 mM did not result in a significant decrease in metabolic activity (Figure 2A), but instead resulted in a dose‐dependent decrease in CD1a^+^ MUTZ‐LCs in the epidermal sheets (Figure 2B). Because 170 mM TiALH was not cytotoxic and also resulted in complete migration of MUTZ‐LCs out of the epidermis, this concentration was used for further experiments.

**FIGURE 3:**
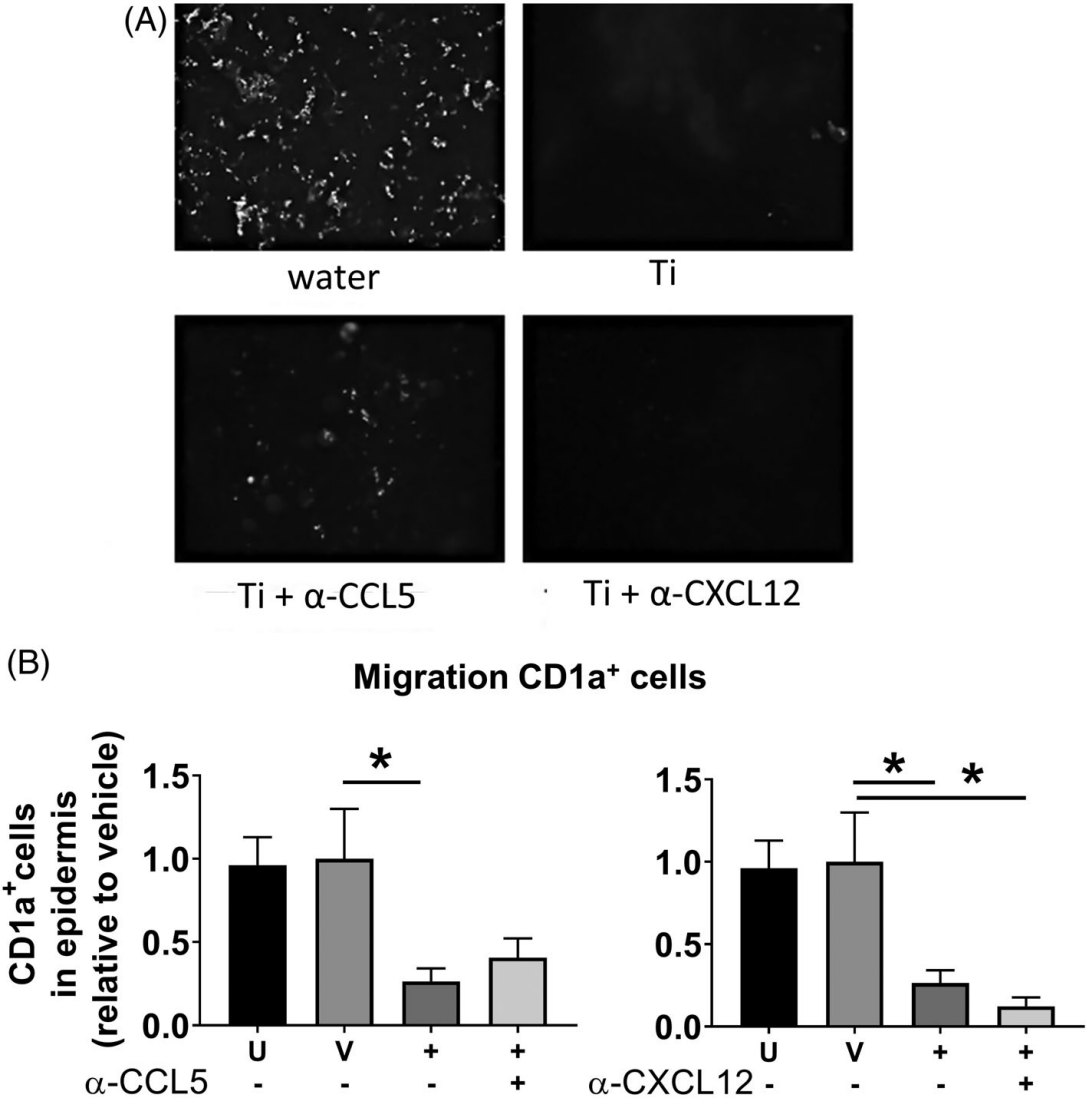
MUTZ‐LC migration out of the epidermis after exposure to titanium(IV) bis(ammonium lactato)dihydroxide is CCL5 dependent. RHS‐LCs were unexposed (U), exposed to H_2_O vehicle (V), or 170 mM TiALH (+) for 24 hours. Chemical exposure was performed in the presence of neutralizing antibodies to CXCL12 (+) or CCL5 (+) or IgG1 isotype control (−). (**A**) Epidermal sheets isolated from RHS‐LCs stained with anti‐CD1a‐PE are shown. Fluorescence intensity (light) shows the presence of MUTZ‐LCs in the epidermal sheets. (**B**) CFSE/CD1a‐PE MUTZ‐LCs in the epidermal sheets were quantified using NIS‐Elements software. Data represent the average of four individual experiments performed in duplicate ± SEM. *P < .05 calculated using the Mann‐Whitney *U* test. Ig, immunoglobulin; LC, Langerhans cell; PE, phycoerythrin; RHS, reconstructed human skin; SEM, standard error of the mean; TiALH, titanium(IV) bis(ammonium lactato)dihydroxide

**FIGURE 4:**
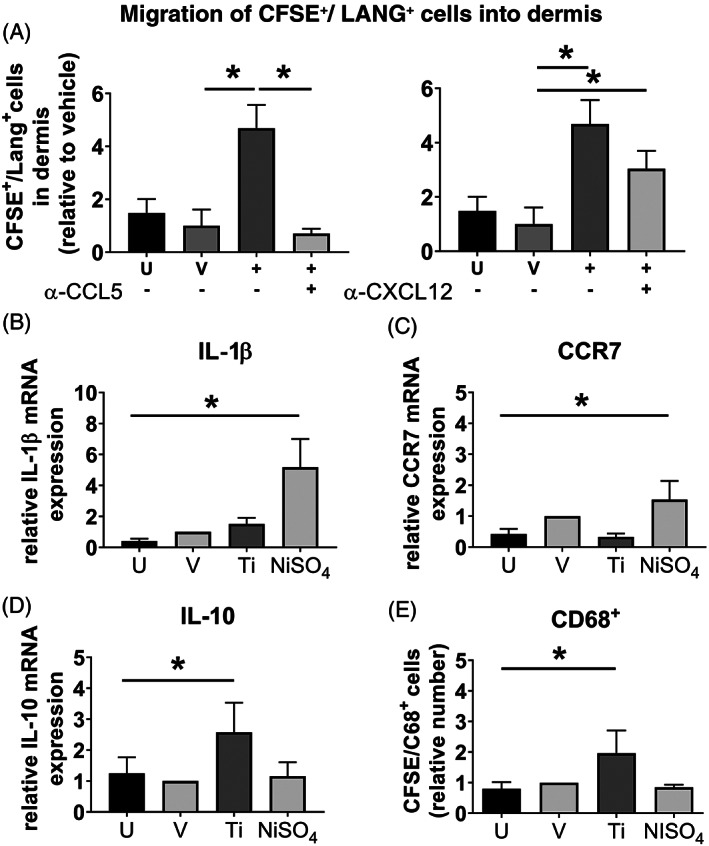
RHS dermis is CD68^+^/IL‐10^high^/CCR7^low^/IL‐1β^low^ after CCL5‐dependent MUTZ‐LC migration. RHS‐LCs were unexposed (U), exposed to H_2_O vehicle (V), 170 mM TiALH (+), or NiSO_4_ (10 mM) for 24 hours. (**A**) Chemical exposure was performed in the presence of neutralizing antibodies to CXCL12 (+) or CCL5 (+) or IgG1 isotype control (−). CFSE/Langerin‐APC fluorescence intensity of MUTZ‐LCs in the dermis was quantified using the CellQuest Pro FACS analysis software. Real time‐polymerase chain reaction shows increased (**B**) IL‐1β and (**C**) CCR7 mRNA after NiSO_4_ exposure, but not after titanium(IV) bis(ammonium lactato)dihydroxide exposure and (**D**) increased IL‐10 mRNA after exposure to titanium(IV) bis(ammonium lactato)dihydroxide but not after exposure to NiSO_4_. (**E**) Increased numbers of viable CD68^+^ cells (flow cytometry) in RHS‐LC dermis after exposure to titanium(IV) bis(ammonium lactato)dihydroxide but not after exposure to NiSO_4_. Data represent the average of four individual experiments performed in duplicate ± SEM. *P < .05 calculated using the Mann‐Whitney *U* test. CFSE, carboxyfluorescein succinimidyl ester; FACS, fluorescence‐activated cell sorting; Ig, immunoglobulin; IL, interleukin; LC, Langerhans cell; mRNA, messenger RNA; NiSO_4_, nickel sulfate; RHS, reconstructed human skin; SEM, standard error of the mean

To determine whether MUTZ‐LC migration was mediated via the sensitizer CXCL12 or the irritant CCL5 mechanism,^32^ the culture medium was next supplemented with neutralizing antibodies against CXCL12 or CCL5 during TiALH exposure (Figure 3). Anti‐CXCL12 had no inhibitory effect on MUTZ‐LC migration, whereas incubation with anti‐CCL5 was able to partially block titanium‐mediated CFSE/CD1a^+^ MUTZ‐LC loss from the epidermis (Figure 3A,B). Because CD1a surface levels on MUTZ‐LCs decrease during irritant‐induced epidermis‐to‐dermis migration,^34^ it could not be concluded whether this was indeed a partial block of migration, whether the chemical concentration used was slightly cytotoxic, or whether the MUTZ‐LCs had decreased their CD1a surface expression upon exposure to titanium. Therefore, CFSE^+^/LANG^+^ cells in the collagen hydrogel of RHS‐LCs were next quantified to confirm migration of MUTZ‐LCs. Exposure to TiALH resulted in approximately five times more CFSE^+^/LANG^+^ cells in the collagen hydrogel compared with vehicle‐exposed RHS‐LCs (Figure 4A). This CFSE^+^/LANG^+^ MUTZ‐LC migration into the collagen hydrogel was completely blocked after incubation with anti‐CCL5. By contrast, after incubation with anti‐CXCL12, titanium‐induced CFSE^+^/LANG^+^ MUTZ‐LC migration into the dermis remained unaffected (Figure 4A). This indeed confirmed that MUTZ‐LC migration out of the epidermis and into the dermis of RHS‐LCs was mediated by the irritant CCL5 pathway and not by the sensitizer CXCL12 pathway.

### 3.3 Migrated MUTZ‐LCs undergo a phenotypic change into a macrophage‐like cell upon titanium exposure

To further identify the phenotype of the migrated LANG+ MUTZ‐LCs within the dermis, the expression of two DC maturation‐related biomarkers (IL‐1β and CCR7)^24^, ^30^ and two macrophage‐related biomarkers (IL‐10 and CD68)^36^, ^40^ was determined. In line with our previous study, the contact sensitizer NiSO_4_ resulted in an increase in IL‐1β and CCR7 mRNA in the dermis of RHS‐LCs,^33^ but not in an increase in IL‐10 mRNA or CD68^+^/CFSE^+^ cells.^34^ However, exposure to titanium did not result in an increase in IL‐1β (Figure 4B) or CCR7 (Figure 4C) mRNA, but did result in an increase in both IL‐10 mRNA (Figure 4D) and CD68^+^ cells^18^ (Figure 4E). These results strongly support an MUTZ‐LC phenotypic change into a macrophage‐like cell upon titanium exposure, in line with RHS‐LCs previously exposed to irritants.^34^

## 4 DISCUSSION

Our results suggest that titanium has irritant rather than sensitizing properties. We show that LC migration into the collagen hydrogel of RHS‐LCs upon topical exposure to TiALH is CCL5 dependent and not CXCL12 dependent, indicating that migration is irritant‐mediated and not sensitizer‐mediated.^31^ This was further supported by the irritant‐mediated phenotypic change of MUTZ‐LC into a macrophage‐like cell,^36^ where we observed that titanium exposure did not result in an increase in IL‐1β or CCR7 mRNA, but by contrast did result in an increase in both IL‐10 mRNA and CFSE^+^/LANG^+^ cells^34^ in the collagen hydrogel of RHS‐LCs. This finding is supported by results from the MUTZ‐LC assay in which four titanium salts were tested. Only TiALH exposure resulted in mild cytotoxicity, and even though a small increase was observed in the expression of maturation biomarkers CD83 and CD86, no increase in CXCL8 secretion was observed. This indicates that the activation of MUTZ‐LCs by TiALH was incomplete because the in vitro exposure led to the upregulation of costimulatory molecules but not to induction of a cytokine response.^41^ Full DC activation (key event 3) in the sensitization pathway is dependent on secondary signals (key event 2) from the tissue environment (eg, KCs) to eventually elicit a T‐cell response.^42^, ^43^, ^44^ Therefore, RHS‐LC was used as a more advanced model to test TiALH, and even in this model, DC activation towards a sensitization pathway was not observed.

As with all studies, our study also has its limitations. All of the titanium salts tested in the MUTZ‐LC assay were soluble in culture medium with the exception of TiO_2_ which remained in suspension. In line with our study, TiO_2_ did not reduce cell viability in vein (human umbilical vein endothelial cell), lung carcinoma (A549), and skin (L929) cells, but did induce reactive oxygen species production.^45^ Only TiALH resulted in mild cytotoxicity at the highest concentration, indicating that in MUTZ‐LC cultures, titanium salts are relatively inert. For this reason only TiALH was used in the RHS‐LC model. Mild cytotoxicity (decrease in metabolic activity) was observed when high concentrations of TiALH were topically applied to RHS‐LCs, indicating that this salt was able to penetrate the stratum corneum. Of note, previously we have shown that none of the four titanium salts were cytotoxic when applied to RHE, therefore scoring as very weak irritant.^18^ This difference in result concerning TiALH (which in this study did show moderate cytotoxicity) can be explained by the extended culture time of RHE (14 days air exposed) compared with RHS‐LCs (10 days air exposed), resulting in RHE having a thicker stratum corneum, and therefore a more competent barrier function than RHS‐LCs. During culture of organotypic skin models, desquamation does not occur, and therefore the stratum corneum increases in thickness with increasing age of the cultures.

TiO_2_ is the most common patch test salt used in the clinic. However, it has been shown that TiO_2_ is not able to penetrate through the stratum corneum to reach the viable skin layers, resulting in false‐negative test results.^14^ Furthermore, our recent retrospective clinical study concluded that TiO_2_ is not the most reliable patch test preparation for the detection of a suspected titanium allergy (TiALH was not included in the test panel).^46^ These findings are in line with our in vitro studies in which we show that TiO_2_ was not able to reduce metabolic activity in RHE nor increase IL‐18 sensitizer biomarker release^18^ and that it showed poor solubility (suspension forming) in the MUTZ‐LC assay. The titanium salt concentrations used in our study can be regarded as being relevant because in our retrospective study on titanium sensitivity, we reported that titanium patch test salts are used at concentrations ranging from 0.08% to 20% depending on the clinical study and the salt used.^46^ Therefore, the concentration of TIALH used in our study was of clinical relevance because we used 170 mM (50 mg/mL, 5%) for topical exposure of the RHS with integrated MUTZ‐LCs. For the conventional submerged MUTZ‐LC monocultures a lower titanium concentration was selected (maximum 1500 μM) to take into account that the barrier property of the stratum corneum was absent, that the TiO_2_ was poorly soluble, and that the TiALH induced mild cytotoxicity already at this concentration. A criteria for the assay is that no more than 25% cytotoxicity occurs if cytokine secretion (CXCL‐8) is a readout.^32^

The question remains that if titanium is not a sensitizer, what could explain the adverse localized “allergic” reactions in titanium implant patients? In the literature the relationship between postoperative complaints in implant patients and titanium allergy is highlighted,^15^, ^47^, ^48^, ^49^ suggesting that true allergy to titanium may exist. However, our retrospective study on titanium hypersensitivity with 468 patients who underwent titanium salt patch testing could not identify titanium‐specific risk factors, nor could a clinical picture after patch testing be identified.^46^ It is possible that, due to the manufacturing process, titanium implants may contain elements (impurities) that have been associated with allergic reactions, such as nickel and palladium.^50^ Previously, it has been demonstrated in a mouse lymph node proliferation assay that titanium nanoparticles (TiO_2_) can modulate chemically induced in vivo dermal sensitization, by acting as an adjuvant to increase the dermal sensitization capacity of a moderate skin sensitizer (eg, 2,4‐dinitrochlorobenzene, DNCB).^51^ Further, it is known that coapplication of an irritant (eg, sodium dodecyl sulfate, SDS) will facilitate sensitization.^52^ Therefore, it is possible that in titanium medical devices with metal sensitizer impurities, titanium may act as an adjuvant. However, this requires further investigation. Taking together the findings by others, our previous findings using the RHE IL‐18 assay,^18^ and this study, we would classify and label titanium as a weak irritant rather than a sensitizer. However, it should be realized that we performed our in vitro assays under sterile conditions. Environmental factors such as the role of the skin and oral microbiome may play an important role in sensitization by influencing directly the epithelial barrier function and by modulating the innate immune system.^53^, ^54^ Indeed, coexposure with bacterial LPS was required to achieve monocyte‐derived DC maturation upon exposure to physiological amounts of metal leachables from dental implants.^55^ To investigate this further, it would be necessary to expose RHS‐LCs to titanium in the presence of a relevant skin or oral microbiome in the future. However, from our present study, we can conclude that the titanium salt TiALH is an irritant rather than a sensitizer, indicating that titanium implant‐related complaints may be due to localized cytotoxicity arising from leachables and corrosion rather than a titanium metal allergy.

## AUTHOR CONTRIBUTIONS

**Charlotte Rodrigues Neves:** Conceptualization; data curation; formal analysis; investigation; methodology; writing‐original draft; writing‐review and editing. **S W Spiekstra:** Data curation; formal analysis; investigation; methodology. **Niels de Graaf:** Conceptualization; methodology; writing‐review and editing. **Thomas Rustemeyer:** Conceptualization; investigation; methodology; writing‐review and editing. **Albert Feilzer:** Conceptualization; funding acquisition; investigation; methodology; project administration; writing‐review and editing. **Cees J. Kleverlaan:** Conceptualization; investigation; methodology; writing‐review and editing. **S Gibbs:** Conceptualization; data curation; formal analysis; funding acquisition; investigation; methodology; project administration; supervision; writing‐original draft; writing‐review and editing.

## CONFLICT OF INTEREST

The authors declare no potential conflict of interest.
